# *Coxiella burnetii* and its risk factors in cattle in Egypt: a seroepidemiological survey

**DOI:** 10.1186/s12917-023-03577-5

**Published:** 2023-01-31

**Authors:** Abdelfattah Selim, Marawan A. Marawan, Abdelhamed Abdelhady, Fahdah Ayed Alshammari, Abdulmohsen H. Alqhtani, Hani A. Ba-Awadh, Isiaka O. Olarinre, Ayman A. Swelum

**Affiliations:** 1 Department of Animal Medicine (Infectious Diseases), Faculty of Veterinary Medicine, Benha University, Toukh, 13736 Egypt; 2 Department of Parasitology and Animal Diseases, National Research Centre, Dokki, Giza, Egypt; 3 Department of Biology, Faculty of Science and Arts-RAFHA, Northern Border University, Arar, 73213 Kingdom of Saudi Arabia; 4 Department of Animal Production, College of Food and Agriculture Sciences, King Saud University, P. O. Box 2460, Riyadh, 11451 Kingdom of Saudi Arabia

**Keywords:** Q fever, *Coxiella burnetii*, Serosurvey, ELISA, Cattle, Nile Delta, Egypt

## Abstract

Animal production is greatly affected by Q fever. As a result of a lack of methodology and financial means to perform extensive epidemiological surveys, the disease's underdiagnosis has proven to be a challenge for effective control. The present study aimed to determine the seroprevalence of *C. burnetii* in cattle raising in four governorates situated at Nile Delta of Egypt and assess the associated risk factors for infection. A total of 480 serum samples were collected from cattle and examined for presence of anti-*C. burnetii* antibodies using indirect ELISA assay. The overall seroprevalence of *C. burnetii* among examined cattle was 19.8%, with the Qalyubia governorate having the highest prevalence. The results of multivariable logistic regression analysis revealed significant association between *C. burnetii* seropositivity and age, communal grazing and/or watering, contact with small ruminants and history of infertility. According to the findings of this work, *C. burnetii* is circulating among cattle living in Nile Delta. It is suggested that adequate hygiene procedures and biosecurity measures should be implemented to limit the transmission of pathogens within cow herds and potential human exposure.

## Introduction

Q fever is a zoonotic disease that affects both humans and animals and is found all over the world with the exception of New Zealand [1]. The causative agent of the disease is *Coxiella burnetii*, an obligate intracellular Gram-negative bacterium belonging to the *Coxiellaceae* family and *Proteobacteria* phylum [2, 3]. *C. burnetii* affects both mammalian and non-mammalian animals [4]. Domestic ruminants, which shed the bacteria mostly through vaginal discharges, milk, urine, and faeces, are considered as the principle reservoirs of *C. burnetii* for human infection [5, 6].

*C. burnetii* primarily infects humans and animals through inhalation of contaminated aerosols or dust, while oral transmission is debatable [7]. In ruminants, the infection is usually asymptomatic. However, reproductive abnormalities such as stillbirth, delivery of weak offspring and abortion are mostly observed in small ruminants, as well as metritis, infertility and mastitis are the most common clinical manifestations in cattle [8, 9].

During an outbreak, some hygienic measures such as removal of aborted materials, manure management, and disinfection of contaminated utensils can help to minimize disease spread. Otherwise, vaccination can decrease the abortion rate and organism shedding, and phase I vaccine is suggested in animals as it is more protective than phase II vaccine [10].

In humans, Q fever can present as an asymptomatic without any clinical signs to acute form with fever, hepatitis and atypical pneumonia or persistent focalized *C. burnetii* infections with signs exhaustion, heart disease and abortion [11].

Many countries have conducted serological surveys to determine the prevalence of *C. burnetii* in domestic ruminants [12, 13]. Serological techniques such as complement fixation test (CFT), immunofluorescence assays (IFAs), and enzyme-linked immunosorbent assays (ELISAs) are used to diagnose Q fever. Because ELISA is more sensitive than IFA, it is more preferred [14]. A few serological investigations on bovine coxiellosis were conducted in several Egyptian locations, focusing on a small number of cows and using various sampling procedures [15, 16].

Therefore, the present study aimed to determine the seropevalence of coxiellosis in cattle in four governorates located in Nile delta of Egypt and evaluate the associated risk factor for *C. burnetii* infection.

## Materials and methods

### Ethical statement

The study followed the Declaration of Benha University and was approved by the Ethics Committee of the Faculty of Veterinary Medicine. All methods were carried out in accordance with relevant guidelines. The owners of the cattle provided informed verbal consent for the samples to be taken. All participants provided informed consent for participation. This study was carried out in compliance with the ARRIVE guidelines.

### Study area

**Fig. 1:**
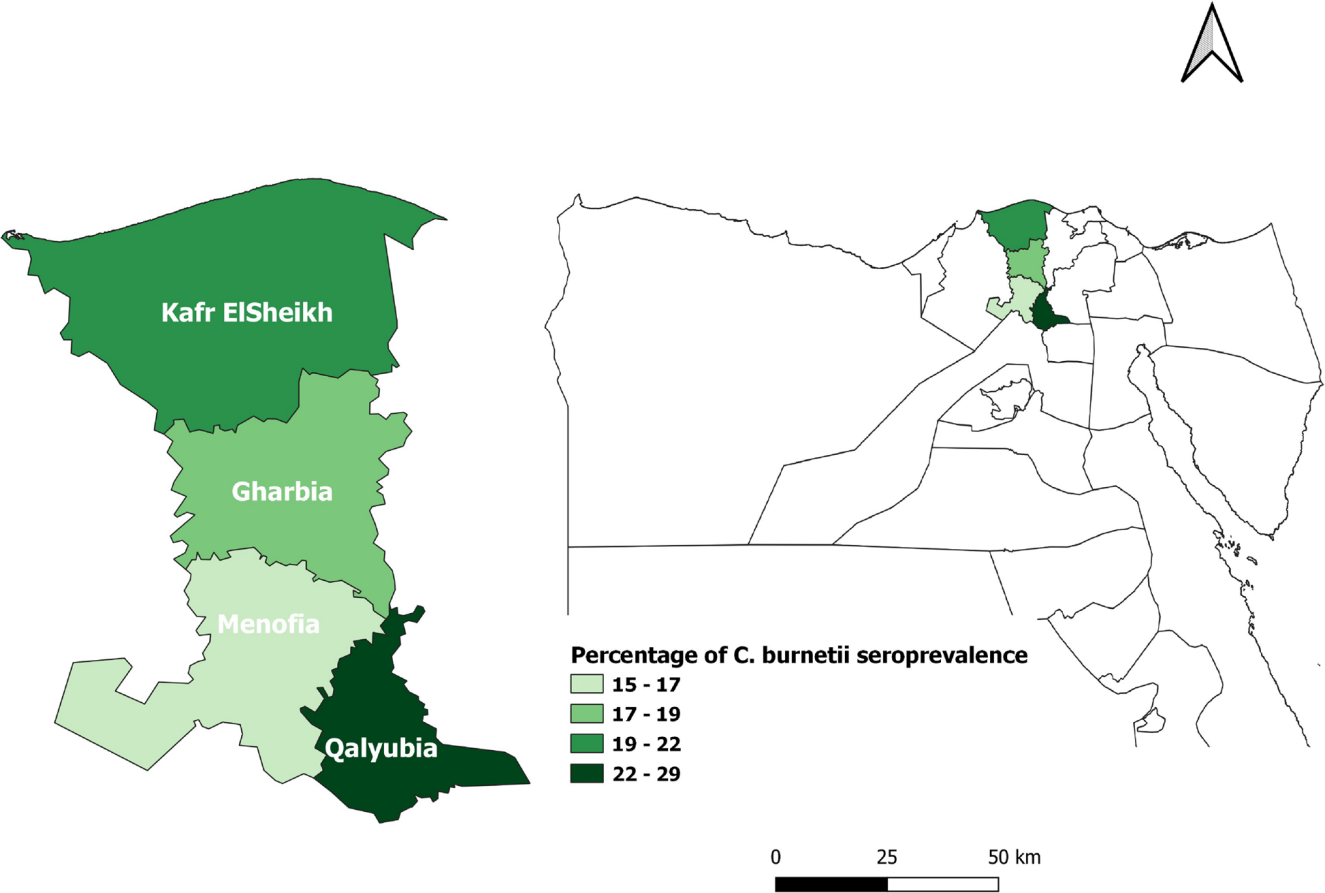
Map of studied governorates in Nile Delta of Egypt

A cross sectional study was performed from January to December 2020 in four governorates (Kafr ElSheikh, Gharbia, Menofia and Qalyubia), situated geographically in Nile Delta of Egypt, Fig. 1. It is a fertile agricultural region and one of the world's greatest river deltas. The Delta, like the rest of Egypt, has a hot desert climate (Köppen: BWh), and as the rest of Egypt's northern coast, which is the driest region in the country, has more moderate temperatures, with high temperature rarely exceeding 31 °C in the summer. In an average year, the delta area receives only 100–200 mm of rain, with the majority of this occurring during the winter months. July and August are the hottest months in the delta, with an average temperature of 34 °C.

### Sampling and data collection

The number of sampled cattle was determined by sample size formula of Thrusfield [17] as following.

$$\mathrm n\hspace{0.17em}=\hspace{0.17em}\left(1.96\right)^2\mathrm p\;\left(1-\mathrm p\right)/\mathrm d^2$$

where n referred to sample size, 1.96 is the Z value for the chosen confidence level (95%), P is the disease prevalence (it was 19.3% as previously reported by Klemmer et al. [15]), and d is the estimated precision at 5%. The calculated sample size was 240 and increased to 480 to increase the precision. A total of 480 blood samples were obtained from randomly selected cattle and transferred in ice box to Veterinary Diagnostic Laboratory, Faculty of Veterinary Medicine, Benha University. The sera were separated by centrifugation at 4000xg for 5 min and kept at -20 °C until serological testing.

On the day of sampling, farmers fulfilled questionnaire with variables related to *C. burnetii* infection including location, sex (male or female), age (2–5, > 5–8 and > 8 years), communal grazing and watering, contact with small ruminants and history of abortion or infertility in the previous year.

### Serological analysis

Each serum sample was tested for antibodies against phase I and phase II antigens of *C. burnetii* using a commercial ELISA ID Screen Q Fever Indirect Multi-species Kit (IDvet, Grabels, France) according to the manufacturer's protocol. According to the manufacturer's internal validation report, this kit has a 100% specificity and a 100% sensitivity for *C. burnetii* detection [18]. The optical density of plates was measured at 450 nm using a spectrophotometer (AMR-100, AllSheng, China), and the results were represented as a percentage of the test sample OD_450_ to the positive control OD_450_ (S/P %). The positive test samples had a S/P % of ≥ 40%, whereas negative samples had a S/P% of less than 30%. The samples with a S/P% between 30 and 39% were doubtful, and were deemed as negative.

### Statistical analysis

The obtained data from serological survey was analysed using SPSS software ver. 24 (IBM, USA). For each variable, a chi-square test was used to perform univariable analysis, and variables with *P* < 0.2 were subjected to multivariable logistic regression analysis. Backward stepwise selection was used to do the multivariable analysis. Statistical significance was determined for all variables with a *P* < 0.05. Hosmer and Lemeshow goodness-of-fit tests were used to evaluate the model's fit [19].

## Results

Ninety-five out of the 480 cattle tested positive for antibodies of *C. burnetii* phase I and phase II antigens, representing 19.8% (95%CI: 16.47–23.59) of the total. The highest seroprevalence was found in Qalyubia governorate (24.2%, 95%CI: 17.39–32.55), while the lowest seroprevalence was observed in Menofia governorate (16.7%, 95%CI: 11.06–24.35), Table 1.

**Table 1:** Univariable analysis of associated variables for *C. burnetii* seropositivity in cattle from Egypt's Nile delta

variable	No of tested samples	No of positive	No of Negative	% of positive	95%CI	Statistic
**Governorates**						
Gharbia	120	21	99	17.5	11.74–25.28	χ2 = 2.664 df = 3 *P* = 0.446
Kafr ElSheikh	120	25	95	20.8	14.53–28.94	
Menofia	120	20	100	16.7	11.06–24.35	
Qalyubia	120	29	91	24.2	17.39–32.55	
Sex						
Male	110	17	93	15.5	9.88–23.36	χ2 = 1.691 df = 1 *P* = 0.193
Female	370	78	292	21.1	17.23–25.52	
Age						
2–5	140	16	124	11.4	7.16–17.76	χ2 = 10.011 df = 2 *P* = 0.007*
> 5–8	215	54	161	25.1	19.79–31.32	
> 8	125	25	100	20.0	13.93–27.86	
Communal grazing area						
No	150	21	129	14.0	9.34–20.46	χ2 = 4.610 df = 1 *P* = 0.032*
Yes	330	74	256	22.4	18.25–27.22	
Shared watering points						
No	120	16	104	13.3	8.37–20.56	χ2 = 4.204 df = 1 *P* = 0.040*
Yes	360	79	281	21.9	17.97–26.5	
Presence of small ruminants						
No	210	25	185	11.9	8.19–16.98	χ2 = 14.629 df = 1 *P* < 0.0001*
Yes	270	70	200	25.9	21.07–31.47	
History of abortion in previous year						
No	435	84	351	19.3	15.88–23.28	χ2 = 0.699 df = 1 *P* = 0.411
yes	45	11	34	24.4	14.23–38.67	
History of infertility in previous year						
No	424	76	348	17.9	14.56–21.85	χ2 = 7.981 df = 1 *P* = 0.005*
Yes	56	19	37	33.9	22.92–47	
Total	480	95	385	19.8	16.47–23.59	

^*^The result is significant at *P* < 0.05

The present findings showed that sex and history of abortion had not significant association with *C. burnetii* seropostivity (*P* > 0.05). Concerning to age, the seropositivity of *C. burnetii* was significantly higher (25.1%) in cattle of median age (> 5–8 years) compared to other age groups. Furthermore, the seroprevalence of *C. burnetii* increased significantly in communal grazing and watering points (22.4% and 21.9%, respectively).

In the light of our findings, the contact with small ruminants and history of infertility in previous years were strongly associated with seropositivity of *C. burnetii* (25.9% and 33.9%, respectively) when compared with other animals, Table 1.

The variables with *P* < 0.2 in univariable analyses were selected for multivariable logistic regression analysis. The findings revealed that age group > 5–8 years (OR = 2.51, 95%CI: 1.34–4.67), communal grazing (OR = 1.43, 95%CI: 0.62–3.32), sharing watering points (OR = 1.55, 95%CI: 0.62–3.92), contact with small ruminants (OR = 2.68, 95%CI: 1.60–4.49) and history of infertility in previous year (OR = 2.61, 95%CI: 1.38–4.94) were identified as potential risk factors for *C. burnetii* infection, Table 2. The final model was better fit (Hosmer and Lemeshow test: χ2 = 6.576; *P* = 0.583).

**Table 2:** Multivariable logistic regression analysis of Risk factors associated with *C. burnetii* seropositivity in cattle from Egypt's Nile delta

Variable	B	S.E	OR	95% C.I. OR	*P* value
Age					0.015
> 5–8	0.918	0.318	2.51	1.34–4.67	0.004
> 8	0.670	0.357	1.95	0.97–3.94	0.061
**Communal grazing area**					
Yes	0.358	0.429	1.43	0.62–3.32	0.405
**Shared watering points**					
Yes	0.440	0.472	1.55	0.62–3.92	0.351
**Presence of small ruminants**					
yes	0.987	0.262	2.68	1.60–4.49	< 0.0001
**History of infertility in previous year**					
Yes	0.961	0.325	2.61	1.38–4.94	0.003

*B* Logistic regression coefficient, *SE* Standard error, *OR* Odds ratio, *CI* Confidence interval

## Discussion

In Egypt, *C. burnetii* was reported to be common in sheep and goats flocks and is recognized to be contributing factor for abortion in sheep, which causes significant economic losses to livestock industry. Nonetheless, there was very little information available on the present state of Q fever in other ruminant species, such as cattle.

In order to gain a better understanding of the current situation of *C. burnetii* infection among cattle in Egypt's Nile Delta, we determined the seroprevalence of *C. burnetii* in cattle reared in these locations and its associated risk factors. In this study, we used the ELISA test rather than other serological tests to investigate *C. burnetii* antibodies in serum samples since it has a higher sensitivity, is quicker, less expensive, easier to execute in labs, and has a larger throughput [20]. ELISA is an indirect diagnostic method that detects prior exposure to *C. burnetii* by identifying specific antibodies; nevertheless, a positive result does not exclude the possibility of a current infection, which necessitates the use of direct diagnostic techniques such as antigen ELISA or PCR [21–24]. The data of current sero-survey prove presence of natural *C. burnetii* infection among cattle in Egypt's Nile Delta, because vaccination of ruminants against coxiellosis is not applied in Egypt [25, 26].

Overall, the seroprevalence of *C. burnetii* in cattle in this study was 19.8%, that is consistent with 19.3% reported in previous epidemiological survey in Egypt except Sinai [15]. Furthermore, the reported prevalence was lower than the 24% found in Beni Suief, Fayoum and Giza governorates [27] and the 50.7% in Assiut governorate, situated at southern Egypt [26]. However, our study's seroprevalence was higher than those reported in Egyptian cattle (13% and 13.2%, respectively) by Nahed and Khaled [28] and Gwida et al. [29]. This disparity in prevalence could be due in part to differing sampling procedures or management issues in these different locations [18].

Many countries have reported bovine coxiellosis with varying prevalence rates. When compared to other serological studies conducted in African countries, our seroprevalence appears to be lower than that reported in Sudan (29.92%) [30] and in Cameroon (31.3%) [31]. In contrast, the present seroprevalence was higher than that reported by Adamu et al. [32], Schelling et al. [33], Kamga-Waladjo et al. [34], Alvarez et al. [21] and Capuano et al. [35], who found seroprevalence of 6.8, 4, 3.6, 6.7, 14.4% in Nigeria, Chad, Senegal, Spain and Italy, respectively. The differences in seroprevalence between locations and countries could be due to a variety of factors, including environmental factors, time of the study, and management practices, all of which could influence on *C. burnetii* transmission [32, 36–42].

Concerning cattle age, seroprevalence of *C. burnetii* increased considerably with age. Higher serorpevalence was reported in the age category of > 5 to 8 years, which was consistent with prior findings of Adamu et al. [32]. A similar findings were reported in camels and small ruminants by Selim and Ali [16] and Rizzo et al. [43]. This could be explained by repeated exposure to pathogen throughout life [42, 44–46].

Interestingly, seropositivity to *C. burnetii* is associated with contact with other herds via shared grazing pastures and/or water sources (*P* < 0.01), what comes in agreement with findings reported by Adamu et al. [32]. It is necessary to highlight the fact that the direct contact with infected cattle increased chance of direct transmission of *C. burnetii*, beside contamination of pastures and watering points [18]. This bacterium, in particular, has a high level of resistance to environmental conditions and can remain infectious for months [47]. Moreover, the vaginal discharges and birth materials or urine, feces and milk of infected animals play a vital role for contamination of environment and dissemination of infection between animals at time of watering or grazing [48, 49].

Furthermore, it was revealed that the prevalence of antibodies against *C. burnetii* significantly increased in cattle raised together with small ruminants. These findings tie in line with findings of Menadi et al. [18], Maurin and Raoult [50], where infection risk and genetic diversity of *C. burnetii* are increased in mixed herds.

From the findings, it is clear that animals with history of abortion or infertility had higher seropositivity for *C. burnetii* in comparison with others, which ties well with findings of Adamu et al. [32] and Menadi et al. [18]. This may raise concerns concerning the fact that gravid ruminants are more susceptible to *C. burnetii* infection than non-gravid ruminants, as well as reproductive disorders caused by bacteria infection [51].

## Conclusion

The current study's findings indicate that *C. burnetii* infection is present and spreading among cattle in Nile Delta of Egypt. As a result, some biosecurity precautions must be implemented, mostly focused on risk factors highlighted in this study, such as restricting contact with infected cattle or small ruminants during feeding and watering, which can prevent bacterium spread and probable transmission to people. In order to better understand and control this disease in Egypt, molecular epidemiological research will be required to obtain precise data on the prevalence of infections caused by *C. burnetii* in animals and humans.

## Data Availability

All data generated or analysed during this study are included in this published article.
